# Tissue-Level Laser-Lok Implants Placed with a Flapless Technique: A 4-Year Clinical Study

**DOI:** 10.3390/ma16031293

**Published:** 2023-02-02

**Authors:** Andrea Spinelli, Fausto Zamparini, Georgios Romanos, Maria Giovanna Gandolfi, Carlo Prati

**Affiliations:** 1 Endodontic Clinical Section, Department of Biomedical and Neuromotor Sciences, School of Dentistry, University of Bologna, 40125 Bologna, Italy; 2 Laboratory of Biomaterials and Oral Pathology, Department of Biomedical and Neuromotor Sciences, School of Dentistry, University of Bologna, 40125 Bologna, Italy; 3 Department of Periodontics and Endodontics, School of Dental Medicine, Stony Brook, NY 11794, USA

**Keywords:** dental implants, flapless surgery, laser-lok, marginal bone level, tapered tissue level implant

## Abstract

Background: The present study aims to analyze the use of Laser-Lok microtextured neck implants placed with a transmucosal surgical approach. The marginal bone level (MBL) and periodontal parameters were evaluated in a cohort prospective 4-year clinical study. Methods: A total of 41 implants were placed in 36 healthy consecutive patients (16 males, 20 females, mean age 60 ± 9 years). Tapered tissue level implants, characterized by a 2.0 mm laser-microtextured neck, were used with a flapless approach. Customized abutments and provisional resin crowns were positioned. Definitive metal–ceramic crowns were cemented approximately 4 months after insertion. Periapical radiographs were taken after 1, 3, 6, 12, 36 and 48 months from implant placement to evaluate MBL. Gingival thickness (thin/thick), plaque score (PS) and bleeding on probing (BoP) were evaluated. Results: After 48 months, all implants were safe from complications. No complications, peri-implantitis, early implant failures or mucositis occurred. The survival rate was 100%. Mean MBL during the follow-up was −0.15 ± 0.18 at T1, −0.29 ± 0.29 at T3, −0.45 ± 0.37 at T6, −0.53 ± 0.45 at T12, −1.06 ± 1.13 at T 36 and −1.10 ± 0.89 at T 48. Implants placed 2–3 months after tooth extraction revealed lower MBL variation when compared to those placed immediately (in fresh extraction sockets) or in completely healed ridges (delayed group). Narrower diameter implants (3.8 mm) showed significantly higher MBL variation when compared to 4.6 diameter implants. Multilevel analysis at T48 revealed that among all the evaluated variables, implant diameter was the factor mostly associated with MBL modifications (*p* = 0.027). Conclusion: This 4-year clinical study supports the use of Laser-Lok implants placed at tissue level with a flapless approach. A limited bone loss during the 48-month follow-up was observed. Periodontal parameters were stable with no sign of inflammation or soft tissue alteration. The use of Laser-Lok implants with transmucosal surgery represents a suitable technique with a minimally invasive approach.

## 1. Introduction

Recent dental implant protocols show substantial variations from those initially designed 20–30 years ago. Those protocols required the submerged positioning of the implant, a 4–6-month healing time and a second surgery to expose the neck before the prosthetic phases [1,2]. 

According to recent studies, bone-level and submerged implant insertion may present a similar outcome in terms of peri-implant bone preservation [3], in particular in non-molar areas [4]. However, some disadvantages must be highlighted, including the need to re-expose the implant emergence before the prosthetic phases, the presence of an implant abutment connection relocated deep in bone tissues and the transmucosal soft tissue tunnel. The presence of a deep transmucosal tunnel was demonstrated to induce a higher risk of peri-implant diseases in the long term, in accordance with a previous study [5]. Similarly, histological studies reported that a deeper implant insertion would result in a greater bone loss during the first months after insertion [6]. Both clinicians and patients are now demanding simplified, less invasive surgical protocols with predictable results [7,8,9] to reduce discomfort and post-operative pain caused by flap elevation and surgery connected with implant placement and exposure. 

Transmucosal implant placement may represent a minimally invasive approach to reduce the number of surgeries after implant placement and does not require a second surgical exposition of the implants [8]. 

New surface treatments (coating techniques, blasting with bioactive fillers) [10] and modifications of implant macro- and microgeometry [11,12,13] lead to the design of minimally invasive surgeries for transmucosal implant placement [8].

The flapless approach may lead to important advantages in hard and soft tissue stability in single edentulous areas. This approach requires only a single low-invasive surgery, minimizes soft tissue trauma and contributes to avoiding a second surgery to expose the implant neck [9,14,15,16].

The implant neck morphology plays an important role in the preservation of marginal bone level (MBL) during the healing phases [8]. Laser-Lok is a computer-controlled laser ablation technique, which creates an implant neck with precision-engineered cell-sized microchannels (size 8 μm). It has been demonstrated that this treatment induces a fibroblast growth that leads to a stable connective tissue attachment, providing an epithelial barrier during initial healing phases [17,18,19]. Histological studies confirmed the possibility of achieving a physical connective tissue attachment to the cell-sized microchannel collar of a dental implant [20].

A recent study evidenced that the laser-ablated cell-sized microchannels of the collar surface may have a positive effect on peri-implant trabecular bone remodeling [21]. The use of an implant neck with this design and morphology may be of great interest concerning tissue-level implants placed with minimal invasive surgery (i.e., the flapless approach). To date, no clinical data are reported regarding this protocol.

This study aimed to investigate clinical and radiographic outcomes of tissue-level implants with a Laser-Lok neck placed tissue-level with a flapless technique. The primary outcome was the analysis of implant survival rates and MBL. The secondary outcome was the analysis of soft tissue inflammation by using bleeding on probing (BoP) and plaque score (PS) indexes. All the implants were followed up at 1, 3, 6, 12, 36 and 48 months.

## 2. Materials and Methods

### 2.1. Study Setting and Patient Selection 

The present investigation was designed in the Endodontic Clinical Section of the Dental School of Bologna University. All patients were recruited in one private dental office.

**Figure 1:**
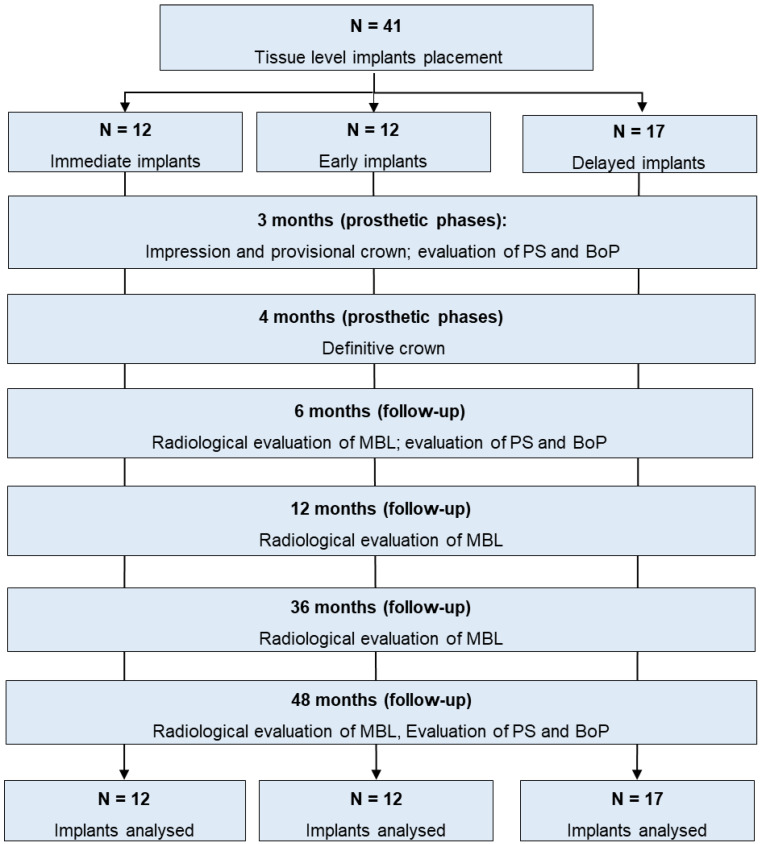
Scheme of the study, research phases and methods.

Patient enrollment started in September 2017 and ended in November 2018 [22]. Patients follow-up had a minimum duration of 48 months. A flowchart of the study protocol is reported in Figure 1.

The patients were considered eligible or non-eligible for inclusion in the clinical protocol based on the following criteria: 

Inclusion criteria:

- 18–75 years of age;
- Requiring a single implant rehabilitation;
- Being able to be included in a hygiene recall program and implant control for at least 4 years;
- Smoking less than 10 cigarettes per day.

Exclusion criteria:

- Medical and/or general contraindications for the surgical procedures (ASA score ≥ 3);
- Poor oral hygiene and lack of motivation (presence of visible plaque on more than 75% of teeth);
- Active clinical periodontal disease in the dentition (probing pocket depth > 4 mm, bleeding on probing in 25% of sites);
- Uncontrolled diabetes mellitus, oncological patients receiving bisphosphonate therapy;
- Alcohol and/or drug abuse as specified in the patient medical anamnesis;
- Pregnancy or lactation period;
- Malocclusion and other occlusal disorder (bruxism, open- and closed bite);
- Lack of minimum crestal bone levels to place a 3.8 × 10 mm implant;
- Post extraction sites requiring guided bone regeneration, biomaterials and membrane insertion.

All patients were treated according to the principles established by the Declaration of Helsinki as modified in 2013. 

Before enrollment, written and verbal information was given by the clinical staff, and each patient gave written consent according to the above-mentioned principles. This report was written according to the Consolidated Standards of Reporting Trials guidelines for reporting clinical trials (STROBE) [23]. Hopeless teeth were radiographically examined to assess the presence/absence of any periapical radiolucency by the clinical team of the Endodontic Clinical Section. 

Choice of the surgical approach and timing of implant placement (immediate, early or delayed according to the timing classification proposed by the Third ITI Consensus Conference) [24] were determined following the principles of “best clinical practice”. Thus, three surgical implant placement timings were defined as follows:

- Immediate post-extraction implant [24]: when the implant was placed into fresh extraction socket immediately after extraction of a root affected by chronic periapical disease and/or seriously damaged hopeless (or fractured) teeth were assigned to this group. Only chronic periapical lesions were present and identified by periapical radiolucency.
- Early implant [24]: when the implant was placed in a healed alveolar bone 8–12 weeks after extraction of the root affected by acute periapical lesion and/or abscess, suppuration and clinical symptoms.
- Delayed implant [24]: when the implant was placed in edentulous mature alveolar bone 10–12 months after the tooth extraction for different reasons (Figure 1).

### 2.2. Surgical Procedures

An implant, characterized by a 2 mm transmucosal part (1.8 mm laser-microtextured surface and 0.2 mm smooth surface in the most coronal part of the implant) was used (Tapered Tissue-level Laser-Lok, Biohorizons, Birmingham, AL, USA). 

One single experienced surgeon performed all surgeries. Before surgery, a careful occlusal and periodontal examination was performed on each patient, including presence of plaque (PS), gingivitis, pocket depth, BoP and radiographic bone loss of all remaining teeth.

Periodontal therapy and oral hygiene training were carried out as needed and as indicated. 

All patients were required to follow a pharmacological regimen that included taking an amoxicillin/clavulanic acid 1 g tablet and applying chlorhexidine di-gluconate 0.2% gel (Corsodyl Gel, GlaxoSmithKline UK, Brantford, UK) twice a day for two days prior to the intervention. All surgical procedures were conducted under local anesthesia with mepivacaine chloral hydrate 30 mg/mL (Carboplyina, Dentsply, Germany). 

A flapless approach was performed for early and delayed timings. The initial drill used to indicate the position, angulation, and depth had a 1.2 mm diameter. The drill passed through the mucosa, cortical bone and cancellous bone under extensive saline irrigation. A twist and calibrated drill at 225 rpm was used and a site of the adequate depth and diameter was created whilst irrigating with a sterile saline solution. The entire rough surface was in the cortical bone, and the divergent surface of the resulting implant was immersed in gingival thickness tissue. 

In case of immediate (in the fresh extraction sockets) implant insertion, an atraumatic flapless root extraction was performed. Inspection of the socket site was carried out and the granulation tissue debrided from the apical portion of the socket. The intra-socket site was prepared with a 1.2 mm drill under generous irrigation following oral bony wall as a guide, followed by a twist and calibrated drill at 225 rpm. The primary implant stability was obtained by anchoring the implant in the remaining apical portion of the socket at least 3 mm beyond the root apex area. No computer-aided guide was used. 

### 2.3. Post-Operative Recommendation

All patients had a surgical periodontal dressing (Coe-Pak^®^, GC, Tokyo, Japan) applied to the wound for one week. Patients were told to follow a soft diet program for one week, rinse three times per day with 0.12% chlorhexidine mouthwash for three weeks and clean their teeth around the Coe-Pak^®^ during the first week and for two weeks after the surgical pack was removed. After that, regular brushing and flossing were allowed.

### 2.4. Prosthetic Rehabilitation

The prosthetic procedures were made four months after insertions in all cases. Polyether impressions (Permadyne and Garant^®^, 3M ESPE, Seefeld, Germany) were obtained using the pick-up plastic customized trays for analogues technique. After 7 days, customized abutments were screwed onto the implants and acrylic temporary single crowns cemented with zinc-oxide temporary cement (Temp Bond^®^, Kerr, Scafati, Italy). After 15 days, a definitive metal–ceramic crown, made by two equally experienced prosthodontists, was positioned on the customized abutment and fixed using a radiopaque polycarboxylate powder/liquid cement (Heraeus Kulzer, Hanau, Germany) with careful attention to prevent any cement overflowing or excess.

### 2.5. Follow-Up Implant Evaluation

#### 2.5.1. Radiographic Assessment of MBL

A paralleling technique with Rinn-holders and analog films (Kodak Ektaspeed Plus, Eastman Kodak Co., Rochester, NY, USA) was used to take intraoral periapical radiographs of all implants at the baseline, 1 month, 3 months, 6 months, 12 months, 36 months and 48 months after implant placement (T48). 

The target-film distance was roughly 30 cm, the exposure period was 0.41 s, the voltage was 70 kV and the intensity was 8 mA. Following the manufacturers recommendations, radiographic development was carried out in a developer unit (Euronda s.p.a., Vicenza, Italy) at standard room temperature (25 °C) with 12 s developing and 25 s fixing times. When not fulfilling the parameters, patients were asked to get a new radiograph. All periapical radiographs were then scanned with a scanner with the following acquisition parameters: resolution 968 dpi and ×20 magnification factor. 

A slide scanner with a resolution of 968 dpi and a magnification of ×20 was used to scan all radiographs. The measurement was calibrated using implants with known lengths and diameters [16,25]. Calibration of brightness and contrast was performed in order to standardize the acquisition of the images.

To assess the MBL change, the crestal marginal bone and the bone–implant contact were examined. Using a scale with 0.1 mm increments, the distance from the reference point (the implant shoulder) to the level of coronal bone-to-implant contact was measured in order to evaluate MBL at the mesial and distal implant surfaces. The implants length and diameter were used to calibrate the measurements of the MBL.

One operator conducted the single-blind radiographic evaluation. A reference set of radiographs with various MBL values and clear instructions was used to calibrate the operator prior to the radiographic evaluation.

#### 2.5.2. Analyzed Variables Related to MBL

MBL was measured and evaluated according to the following variables:

- Pre-operative parameters: gender, implant location, time of implant placement;
- Intraoperative parameters: implant diameter;
- Post-operative parameters: gingival thickness.

### 2.6. Clinical Periodontal Parameters

PS and BoP [7] were monitored around the implant restoration and in correspondence with adjacent teeth at 3 months (T3), 6 months (T6), 12 months (T12) and 48 months (T48).

Around the implant restorations and on adjacent teeth, four sites (mesial, distal, buccal, and oral) were evaluated for PS. The results were expressed as a dichotomous score (0 = no visible plaque at the soft margin; 1 = visible plaque at the soft margin). 

BoP was assessed at four sites (mesial, distal, buccal, and oral) [7] around the implant restorations and on adjacent teeth, and a dichotomous score (0 = no bleeding; 1 = bleeding) was given.

During the surgical operations, the gingival thickness around the implants and their corresponding mesial neighboring teeth was identified. An endodontic file was used to penetrate the soft tissue three millimeters apical to the gingival edge (K-file Nr. 20; Dentsply-Maillefer, Tulsa, OK, USA). According to the mean registered value, the gingival phenotype was classified as thick (soft tissue thickness > 2 mm) or thin (soft tissue thickness 2 mm) [26].

### 2.7. Statistical Analysis

Stata 17.1 (StataCorp, College Station, TX, USA) was used to perform all statistical analysis.

The skewness and kurtosis indexes were used to measure the distribution of the samples. Due to the normal distribution of data, linear regression models were fitted to evaluate the existence of any significant difference regarding the evaluated parameters, times (T1, T3, T6, T12, T36, T48) and the interactions between parameters and time. The aforementioned regression models were calculated using a generalized estimating equation approach in consideration of the correlation of the data caused by the presence of many implants per subject. Using a reliable variance–covariance estimator, we modified the estimates of the standard errors of the coefficients and the confidence intervals. 

The connection between MBL at 48 months and the analyzed variables was assessed using a multiple linear regression with stepwise selection.

## 3. Results

A total of 41 implants were placed in 36 consecutive patients (16 males, 20 females, mean age 60 ± 9 years). No complications, peri-implantitis, peri-implant bone necrosis, early implant failures or mucositis occurred, and the survival rate was 100%. No drop-out has been reported. After 48 months, all implants were safe from complications. 

MBL according to operative parameters is reported in Table 1. 

MBL according to gender and location did not reveal statistically significant differences up to 48 months (*p* > 0.05). 

Differently, some statistically significant differences were observed when considering implant placement timing, implant diameter and gingival thickness.

Implants placed 2–3 months after tooth extraction revealed lower MBL variation when compared to those placed immediately (immediate implants placed in fresh extraction sockets) or in completely healed ridges (delayed group), with a mean bone gain at T1. These values were lower at all evaluation times, but only from T36 were the differences statistically significant.

Implant diameter was found to significantly affect MBL. Narrower diameter implants (3.8 mm) showed significantly higher MBL variation from T3 to T48 when compared to 4.6 diameter implants. At T48, the greatest MBL variations were observed for 3.8 mm implants (mean MBL was 1.43 ± 1.01).

Gingival thickness significantly affects MBL during pre-load. Implants surrounded by a thick biotype showed lower MBL variations when compared to thin tissues. In the post-loading period, the differences decreased, with non-statistical differences at T12 and T48.

Multilevel analysis at T48 revealed that among all the evaluated variables, implant diameter was the factor mostly associated with MBL modifications (*p* = 0.027) (Table 2). 

BoP and PS around implants is reported in Table 3.

Low BoP values were observed at T3, percentages of bleeding sites were 4.7 ± 4.0 (range 0–9.7%) and 4.05% (range 0–9.7%). These percentages were similar at T48 and the mean value was 4.42% (0–6.5%). The mesial site was the most affected.

PS showed a similar trend: sites with plaque accumulation were 8.1% (range 0–12.9%) and 5.2% (range 0–9.7%) at T3 and T6. The most affected areas were the mesial and the buccal sites, while in the oral site no difference was observed. Similar results were observed at T48.

**Figure 2:**
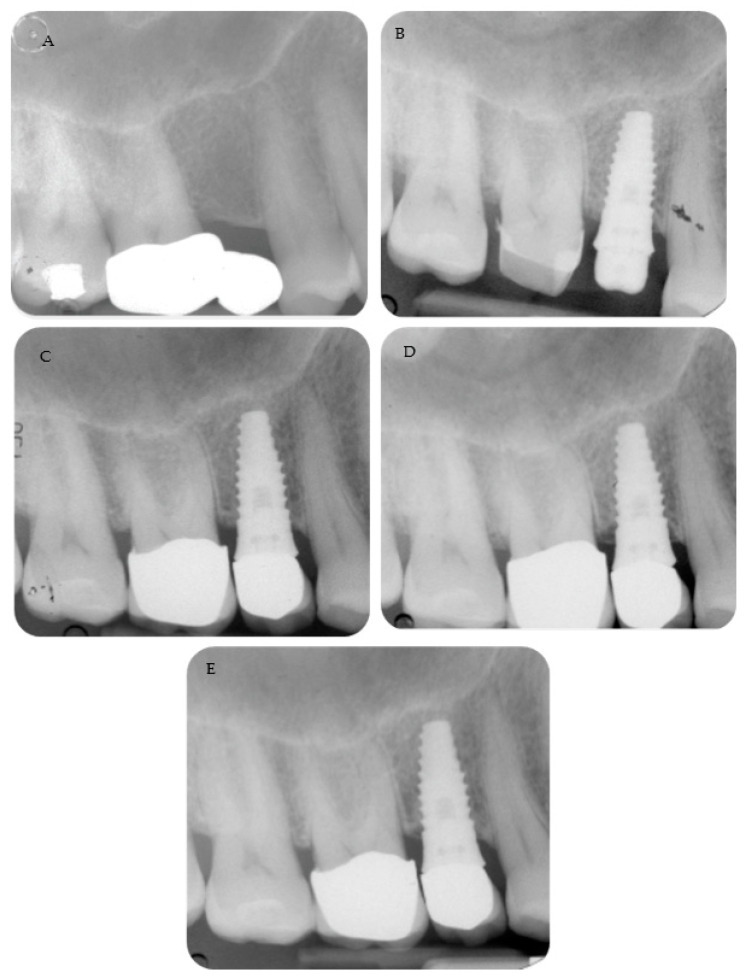
A 4.6 mm tissue-level implant was placed with a flapless approach. (**A**) Pre-operative radiograph. (**B**) Periapical radiograph shows the abutment screwed and the cemented provisional crown. Follow-up at 3 months. (**C**) Follow-up at 12 months with the metal ceramic crown cemented on the abutment. MBLs were stable at (**D**) 36 months and (**E**) 48 months, with limited bone loss.

**Figure 3:**
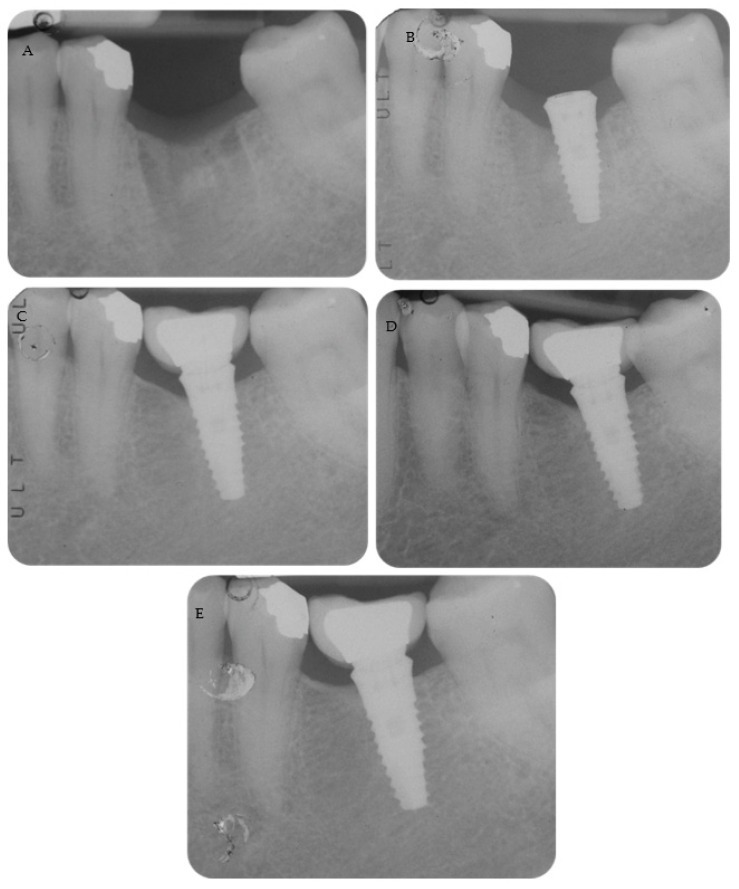
(**A**) Early timing placement to rehabilitate a single edentulous ridge. Tooth was extracted 2 months before for a root fracture. (**B**) Implant placement with a flapless approach. Please note that the neck was positioned at tissue level. (**C**) Periapical radiograph after abutment and definitive crown cementation. (**D**) Follow-up at 36 months and (**E**) 48 months showed MBL stability around the implant.

Periapical radiographs reporting two treated cases are shown in Figure 2 and Figure 3.

## 4. Discussion

In the present study, tissue-level implants with a laser-microtextured neck were placed with a flapless technique with the cover screw exposed. Our findings revealed hard tissue stability and limited MBL during the 4-year period. 

The valid periodontal parameters and MBL observed during the early phase after placement and during the 4-year evaluations support the use of a Laser-Lok surface for an implant placed at tissue-level. Soft tissue morphology and mucosa integrity proved sufficient to avoid bacteria penetration and to induce fibroblast attachment and a correct vascular environment for bone formation and a stable MBL. 

The result of our investigation is supported by a recent clinical study that compared the expression of pro-inflammatory cytokines in peri-implant crevicular fluid of implants placed at bone level versus tissue level [27]. Bone-level implants demonstrated, after 5 years, a higher level of inflammatory cytokines than tissue-level implants [27].

A recent review reported that the fibro-collagenous attachment around the microgrooved neck might stabilize the bone and reduce crestal bone resorption [28]. Previous in vitro investigations revealed osteoblast attachment on the laser-microtextured neck and cell alignment along the microgroove directions [29]. An animal model also demonstrated a direct connective tissue attachment to the microgrooved surface where the fibers were mostly disposed perpendicular to the implant surface [30].

Clinically, these aspects may also induce less incidence of peri-implant inflammation around the transmucosal portion. A recent 5-year retrospective multicenter study reported a lower incidence of peri-implant mucositis on laser-microtextured neck implants, suggesting lower pathological bacteria concentration compared to non-laser implants with the implant design [19].

In this study, different clinical operative parameters were analyzed, including implant placement timing, soft tissue thickness and implant diameters. 

Interestingly and unexpectedly, neither timing nor soft tissue thickness parameters showed any significant differences at 48 months from insertions. Other studies that consider implants characterized with a transmucosal neck [31] or platform-switch implants [16] found different results. In this context, the neck morphology and the laser-microtextured design could have influenced the clinical outcomes.

Interestingly, implant diameter was the factor mostly associated with MBL variation. A wider implant diameter (4.6 mm in this case) better preserved bone marginal morphology and crestal level, while a narrower diameter implant (3.6 mm) revealed greater marginal bone loss. Implant diameter was selected before placement in accordance with the bone crest morphology in healed crest (early and delayed implants) or in accordance with the residual bone in the post-extractive sites (immediate implants).

As observed in Table 2, 3.6 mm diameter implants showed the greatest bone loss at T48. Stress values affecting the crestal cortical bone can influence peri-implant crestal bone resorption. Wider implant diameters may reduce mechanical stress on crestal bone, directly influencing MBL. Some studies reported a higher risk of prosthetic complications for narrow diameter implants, including abutment and implant fracture, screw loosening or fracture and ceramic fracture, which may occur in the long term [32,33]. In the present study, none of these complications occurred.

The proposed protocol allowed the management of dental implant insertion, impression techniques and prosthetic restorations with limited surgical trauma, limited bone loss and no complications. 

A relatively high mean age of patients was observed in this study (mean age 60 ± 9 years). Patients with older age often require minimally invasive protocols including flapless surgeries, one-stage surgery, immediate placement/immediate loading and the use of short and narrow diameter implants [34,35].

All provisional and definitive crowns were cement-retained. Temporary zinc–oxide eugenol-based cement was selected for provisional restorations, as enough retention on the neck has been observed (with no presence of de-cemented crowns). Definitive rehabilitations were cemented with a polycarboxylate-based cement, which made them easier to remove during setting time and less irritating than methacrylate-based definitive cements [36]. According to a review, no significant differences were observed when comparing single implant restorations between cement-retained and screw-retained crowns [37]. Moreover, in the tissue-level technique, the risk for excess cement retention under soft tissue levels was reduced, as the implant abutment connection is located more coronally than in conventional bone-level implants [37].

The limitation of this study may be represented by the relatively low number of patients. In the future, impression techniques must be obtained via a 3D scanner, and the preparation of abutment design may be affected by the new digital technique and workflow. Further studies with a larger sample size and at a longer follow-up should validate the flapless technique with tissue-level implant placement that is proposed here [38,39]. The lack of complications may be influenced by patient selections and by strict compliance with the protocol.

## 5. Conclusions

The use of Laser-Lok implants placed at tissue level using a flapless technique is supported by this four-year clinical research. During the 48-month follow-up, minimal bone loss and stable marginal bone levels were seen. These values were in line with those reported in the literature. This transmucosal flapless technique allows a soft tissue healing as demonstrated by periodontal parameters that were stable during the 4 years of follow-up. No signs of inflammation or soft tissue alteration around the laser-microtextured neck were observed. The wider diameter implant seems to preserve bone levels.

## Figures and Tables

**Table 1:** MBL (Mean ± SD) of the placed implants evaluated at 1 and 3 months (pre-loading) and at 6,12,36 and 48 months (post-loading) from implant insertion. Different superscript letters represent statistically significant differences in the same horizontal row (capital letters among times) or in the same column (small letters for each parameter). *p*-value was set at 0.05.

Parameters			Pre-Loading		Post-Loading			
		n	T_1_	T_3_	T_6_	T_12_	T_36_	T_48_
Gender	Males	17	+0.12 ± 0.22 ^aA^	−0.27 ± 0.45 ^aA^	−0.42 ± 0.39 ^aB^	−0.52 ± 0.39 ^aC^	−0.90 ± 0.48 ^aB^	−1.03 ± 0.49 ^aB^
	Females	24	−0.18 ± 0.20 ^bA^	−0.31 ± 0.33 ^aB^	−0.47 ± 0.50 ^aC^	−0.54 ± 0.38 ^aC^	−1.20 ± 1.13 ^bC^	−1.16 ± 1.10 ^aC^
Implant location	Maxilla	18	−0.12 ± 0.20 ^aA^	−0.28 ± 0.32 ^aA^	−0.47 ± 0.40 ^aB^	−0.61 ± 0.78 ^aB^	−0.92 ± 1.13 ^aC^	−1.08 ± 1.01 ^aC^
	Mandible	23	−0.19 ± 0.21 ^aA^	−0.31 ± 0.35 ^aB^	−0.43 ± 0.41 ^aB^	−0.47 ± 0.39 ^aB^	−1.16 ± 1.19 ^aC^	−1.12 ± 1.16 ^aC^
Implant placement timing	Immediate	12	−0.20 ± 0.22 ^aA^	−0.37 ± 0.35 ^aA^	−0.47 ± 0.45 ^aB^	−0.53 ± 0.12 ^aB^	−1.06 ± 1.11 ^aC^	−1.01 ± 1.01 ^aC^
	Early	12	+0.13 ± 0.24 ^bA^	−0.31 ± 0.38 ^aB^	−0.31 ± 0.43 ^aB^	−0.42 ± 0.41 ^aB^	−0.69 ± 1.18 ^bB^	−0.63 ± 0.89 ^bB^
	Delayed	17	−0.19 ± 0.22 ^aA^	−0.45 ± 0.35 ^aB^	−0.54 ± 0.40 ^aC^	−0.68 ± 0.41 ^aC^	−1.29 ± 1.13 ^aC^	−1.45 ± 1.10 ^aC^
Implant diameter	3.8 mm	14	−0.23 ± 0.10 ^aA^	−0.49 ± 0.33 ^aB^	−0.73 ± 0.40 ^aB^	−0.89 ± 0.38 ^aB^	−1.39 ± 1.13 ^aC^	−1.43 ± 1.01 ^aC^
	4.6 mm	27	−0.12 ± 0.21 ^aA^	−0.19 ± 0.34 ^aA^	−0.29 ± 0.41 ^bB^	−0.35 ± 0.38 ^bB^	−0.64 ± 1.16 ^bC^	−0.64 ± 1.12 ^bC^
Gingival thickness	Thin	23	−0.18 ± 0.20 ^aA^	−0.36 ± 0.33 ^aA^	−0.45 ± 0.38 ^aAB^	−0.57 ± 0.38 ^aB^	−1.09 ± 1.13 ^aC^	−1.27 ± 1.11 ^aC^
	Thick	18	−0.10 ± 0.21 ^aA^	−0.14 ± 0.35 ^aA^	−0.44 ± 0.45 ^aB^	−0.45 ± 0.47 ^aB^	−1.01 ± 1.15 ^aC^	−1.01 ± 1.02 ^aC^
Total		41	−0.15 ± 0.18 ^A^	−0.29 ± 0.29 ^A^	−0.45 ± 0.37 ^B^	−0.53 ± 0.45 ^B^	−1.06 ± 1.13 ^C^	−1.10 ± 0.89 ^C^

**Table 2:** Multilevel mixed-effects logistic regression exploring factors associated with MBL at 48 months.

Parameters	Coefficient	Robust Std. Err.	*p*	[95% Conf. Interval]	
Gender	0.2609	0.2775	0.347	−0.282	0.804
Implant location	−0.1749	0.3525	0.62	−0.865	0.516
Implant placement timing	0.2711	0.2018	0.179	−0.124	0.666
Implant diameter	−0.7855	0.3552	0.027	−1.481	−0.089
Gingival thickness	0.2611	0.4551	0.567	−0.632	1.154

**Table 3:** Peri-implant parameters around implant restorations after provisional and definitive load. Values are expressed as percentages.

Plaque Score (%)							Bleeding on Probing (%)					
	T_3_		T_6_		T_48_		T_3_		T_6_		T_48_	
	0	1	0	1	0	1	0	1	0	1	0	1
Mesial	87.1	12.9	90.3	9.7	87.1	12.9	90.3	9.7	90.3.	9.7	93.5	6.5
Distal	93.5	6.5	94.4	5.6	93.5	6.5	93.5	6.5	100	0	94.4	5.6
Buccal	87.1	12.9	94.4	5.6	94.4	5.6	90.3	9.7	93.5	6.5	94.4	5.6
Oral	100	0	100	0	100	0	100	0	100	0	100	0

## Data Availability

Not applicable.
